# Designing a Unique Medical Student Workshop to Provide Early Exposure to Careers in Gastroenterology and Hepatology

**DOI:** 10.7759/cureus.84135

**Published:** 2025-05-14

**Authors:** Smruti Rath, Brijen Shah, Bhavana B Rao

**Affiliations:** 1 Internal Medicine, Icahn School of Medicine at Mount Sinai, New York, USA; 2 Division of Gastroenterology, Icahn School of Medicine at Mount Sinai, New York, USA

**Keywords:** career advising, gastroenterology, medical education, panel, simulation

## Abstract

Background

Medical students have limited knowledge of the subspecialties and procedural components within Gastroenterology (GI)/Hepatology (Hep). We crafted an interactive session to provide medical students with a broader understanding of the career paths in GI/Hep through perspectives from faculty and simulations.

Methodology

We conceptualized and organized the workshop in March 2024. The first half consisted of a panel discussion involving seven eminent institutional GI/Hep faculty members. The second part offered hands-on stations involving commonly performed GI/Hep procedures. These included endoscopy, paracentesis, capsule endoscopy, inflammatory bowel disease (IBD) therapies, and an esophageal manometry catheter with readings. Pre- and post-surveys were used to assess the impact of the workshop on participants’ interest and understanding of the field. Surveys were reviewed by multiple GI/Hep faculty members for accuracy and appropriateness. The event was advertised via email to all medical students studying at our institution.

Results

Approximately 600 students were emailed, with 46 expressing interest and 30 students attending the workshop. Of the 30 students, 15 (50%) and 14 (47%) students completed the pre- and post-surveys, respectively. A lack of prior exposure to gastroenterology/hepatology outside the classroom was reported by 60% (15 respondents), and 75% (15 respondents) expressed interest in having a procedural component in their future careers. Participants were asked to rank their familiarity with different domains within GI/Hep, with overall improvement in mean familiarity noted after the event. Student comments were overwhelmingly positive, and 93% (14) of the students reported that they were extremely satisfied with all aspects of the workshop.

Conclusions

Our results demonstrated that this hybrid panel and simulation workshop improved medical students' understanding of GI/Hep, and that forums such as these can be valuable to provide early exposure and aid students in career planning.

## Introduction

Clinical rotations occur in the latter half of medical school, and hence, first- and second-year medical students typically have limited exposure to clinical applications of their lecture-based learning [1]. While core clinical clerkships provide exposure to general medicine and surgery, students often do not have exposure to procedural subspecialties, such as Gastroenterology (GI)/Hepatology (Hep), until late in medical school via electives. Additionally, early medical school learners are taught outside the clinical setting. This limits their interaction with residents, fellows, and attending physicians during a critical time in the exploration of specialty preferences and mentorship building. An opinion piece by a medical school graduate emphasized the importance of mentorship and panel discussions in aiding in residency and specialty selection, but also commented on gaps in exposure to career planning after residency [2].

One method of increasing these interactions includes panels for question-and-answer sessions as part of educational curricula or career fairs. One study discussed the utilization of a faculty panel to introduce medical students to academic medicine and revealed mostly positive feedback [3]. Most feedback from students highlighted their interest in hearing different perspectives on each faculty member’s journey to their respective positions and reassurance from hearing personal anecdotes about the panelists’ challenges [3].

There are very few educational initiatives published in the literature that focus on the sub-specialty of GI/Hep for undergraduate medical education (UME). One group implemented a simulation-based program to teach medical students GI/Hep topics. Student responses revealed increased appreciation of the relevance of topics taught in the classroom [4]. While simulation learning poses various challenges, such as the need for significant preparation, support of faculty, and engagement of participants, it offers several benefits, including active learning with real-time feedback and adaptability to different scenarios [5]. When done successfully, it can bridge the gap in UME between classroom education and clinical clerkships.

To address the above shortcomings in our current UME offerings, we crafted a novel workshop for medical students to gain exposure to the medical and procedural specialty of GI/Hep. This workshop aimed to facilitate interaction with fellows and faculty, provide examples of clinical applications of their basic science learning in GI/Hep, expose them to various sub-specialties and procedures within GI/Hep, and provide them with a perspective on careers in the field.

## Materials and methods

This workshop was conceptualized and implemented with the input of gastroenterology specialists involved in medical education and quality improvement at our institution. Leaders of the student GI/Hep interest group were also consulted while developing the session. We crafted a two-hour session, open to all medical students, comprised of an interactive question-and-answer panel followed by a simulation-based workshop.

Six hundred students were emailed across all four medical school classes at our institution. The email contained a description of the format of the workshop, an eye-catching advertising flyer, and a Google sign-up form, which also served as their consent to participate in the workshop and surveys. This form also included an option for students to submit questions for the workshop's initial panel portion. Two re-sends were sent via email to all students to sign up, and a final reminder email was sent to those who signed up to participate.

In the first part of the workshop, the faculty panelists represented a wide range of focuses within GI/Hep, including but not limited to: advanced endoscopy, IBD, motility disorders, irritable bowel disease, colon cancer screening, medical education, quality improvement, transplant hepatology, and women’s health in GI/Hep. Questions were adapted from student submissions and questioned panelists on specific traits of their chosen subspecialty, individual work life routines, insights gleaned from their professional journey, and advice for medical students. Audience questions were also accepted during the session. After completing this panel, students proceeded to the second part of the workshop, which offered hands-on experiences across six stations where students were asked to spend 10 minutes each. Each station was supervised by a fellow and/or resident facilitator who demonstrated simulations that students subsequently attempted. Students could practice performing paracentesis on a simulation mannequin and using an endoscope to simulate techniques in endoscopy. Faculty and fellows also helped explain various devices and samples used in other aspects of GI/Hep, such as capsule endoscope demonstrations, esophageal manometry catheters with readings, endoflip readings, and samples relating to IBD therapeutics.

A pre- and post-survey design was implemented to assess the workshop’s impact on student understanding of and interest in GI/Hep (Appendices A-B). Both surveys were reviewed and edited by GI/Hep specialists with an added niche in medical education. Surveys were created on Google Forms. Question formats included multiple choice, 5-point Likert scales, and free response. Students were provided with the pre-survey before the workshop via email, and the post-survey via QR code at the end of the session and via email.

Results were analyzed both quantitatively and qualitatively. Responses on a 5-point Likert scale were analyzed as ordinal data using the Mann-Whitney U test. Related questions were grouped as a series and compared as interval data using a paired t-test. An alpha level of 0.05 was used for determining significance. All survey responses were voluntary and anonymous.

## Results

Forty-six students expressed interest in attending the workshop through the online sign-up form. Of these, 18 (40%) were first-year medical students (MS1s), 20 (43%) were second-year medical students (MS2s), 4 (9%) were third-year medical students (MS3s), and 4 (8%) were fourth-year medical students (MS4s). Of these students, 30 attended the workshop. Additionally, six internal medicine residents and six GI/Hep fellows participated as volunteers.

The workshop started with a lively discussion between the seven panelists and students in the audience. Questions had been solicited from the students beforehand, and additional questions were brought up by the audience during the session. Topics that were addressed included pathways in selecting each panelist’s respective professional domains, scope and balance of clinical and academic responsibilities, work-life balance, and guidance on finding scholarly pursuits. Following the panel, students were divided into six groups to rotate through the various stations. At the paracentesis station, students practiced ultrasound and paracentesis catheter insertion on a mannequin. At the endoscopy station, students manipulated an endoscope in a simulator to learn basic techniques of maneuvering the scope. They were also able to try tools such as biopsy forceps to grasp foreign objects. At other stations, they held a video capsule, saw the images it generates, examined a manometer catheter, and reviewed its reports. At the IBD station, students used simulated biologic medication injections and learned concepts about IBD therapeutics.

**Figure 1:**
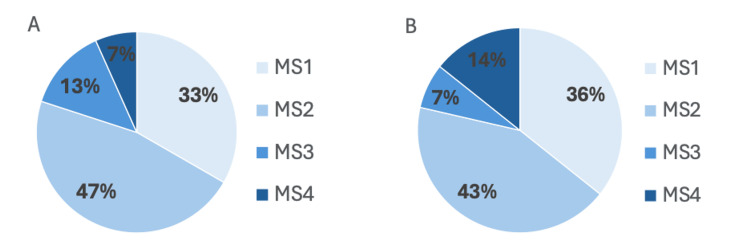
Distribution of training levels of medical students who responded to the (A) pre-workshop survey (n = 15) and (B) post-workshop survey (n = 14).

A total of 15 students (*N *= 30, 50%) and 14 students (*N* = 30, 47%) completed the pre- and post-surveys, respectively. Among those who completed the pre-survey, 5 (33%) were MS1s, 7 (47%) were MS2s, 2 (13%) were MS3s, and 1 (7%) was an MS4 (Figure 1). Among the post-survey respondents, 5 (36%) were MS1s, 6 (43%) were MS2s, 1 (7%) was an MS3, and 2 (14%) were MS4s (Figure 1). Pre-survey responses revealed gaps in exposure to practical applications of gastroenterology and hepatology (GI/Hep) as a career. Of the respondents, 15 (60%) indicated no exposure to GI/Hep outside of the classroom, and 15 (75%) expressed interest in having a procedural component in their career.

All students who completed the pre-workshop survey felt that medical school training should incorporate more networking events and career exposure during the MS1/MS2 years.

**Figure 2:**
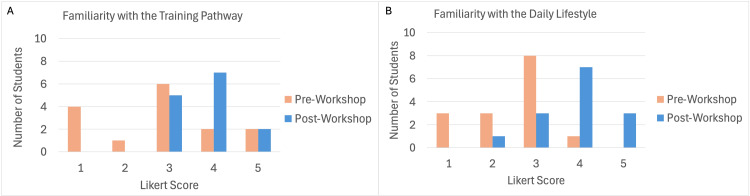
Student familiarity with (A) the training pathway and (B) daily lifestyle of a gastroenterology/hepatology specialist before and after the workshop (1 = not familiar, 5 = completely familiar).

Students were then prompted to rate their familiarity with various domains in GI/Hep with the following 5-point Likert scale: 1, not at all; 2, slightly; 3, moderately; 4, considerably; 5, completely (Table 1). Average familiarity across all domains significantly increased after the workshop by 28.6% (2.1 vs. 2.7; *P* < 0.01). Individual categories with significant increases in familiarity were general endoscopy (2.3 vs. 3.1; *P* = 0.04), advanced endoscopy (1.6 vs. 2.6; *P* = 0.01), and neuro-gastroenterology (1.7 vs. 2.6; *P* = 0.02) (Table 1). Similarly, students were asked to rate their familiarity with the training pathway to become a specialist in GI/Hep and the daily lifestyle in these career paths. Survey responses revealed significant increases in the number of students reporting being *considerably* or *completely* familiar with the training pathway (15, 27% vs. 14, 64%; *P* = 0.04) and daily lifestyle (15, 7% vs. 14, 71%; *P* < 0.01) (Figure 2).

**Table 1:** Pre- and post-workshop familiarity with domains in GI/Hep. Students’ mean familiarity with various domains in GI/Hep with the following scale: 1, not at all; 2, slightly; 3, moderately; 4, considerably; 5, completely. Responses on the 5-point Likert scale were analyzed on the pre- and post-surveys as ordinal data using the Mann-Whitney U test. Related questions were grouped as a series and compared as interval data using a t-test. Results were significant if *P* < 0.05. GI/Hep, Gastroenterology/Hepatology

	Pre-workshop survey (*n* = 15)	Post-workshop survey (*n* = 14)	Test statistic (*U*-value)	*P*-value
General endoscopy	2.3	3.1	56.50	0.04
Advanced endoscopy	1.6	2.6	47.00	0.01
Neuro-gastroenterology	1.7	2.6	50.00	0.02
Inflammatory bowel disease	2.7	2.9	88.00	0.47
Liver medicine	2.1	2.6	72.00	0.16
Transplant hepatology	1.9	2.6	65.00	0.09
Hereditary cancer syndromes	2.0	2.4	81.00	0.30
Medical education	2.4	2.8	81.50	0.32
Microbiome research	2.4	2.4	103.50	0.97
Overall (mean)	2.1	2.7	-4.71 (*t*-value)	<0.01

**Figure 3:**
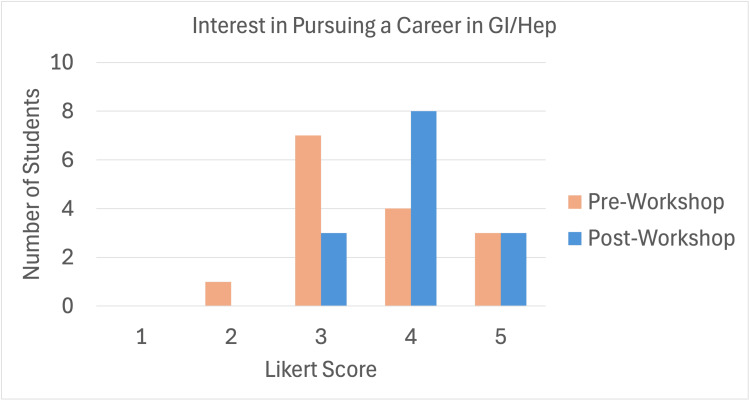
Students' interest in pursuing a career in Gastroenterology/Hepatology (GI/Hep) before and after the workshop (1 = not interested, 5 = completely interested).

There were several other questions posed to students to gauge the impact of the workshop. Students were asked to choose their level of interest in pursuing a career in GI/Hep on a 5-point Likert scale. Pre- and post-survey data revealed a non-significant increase in ratings of considerably and completely interested in GI (15, 7%, vs. 14, 79%; *P* = 0.21) (Figure 3). Following the workshop, 14 (85.7%) respondents stated that the session changed their perception about the field of GI/Hep. Overall, student feedback was overwhelmingly positive, and 93% (*N* = 14) of the respondents reported that they were extremely satisfied with all aspects of the workshop. Free response comments focused on changes in perception of the culture of the field, interest in the faculty perspectives, and newfound knowledge of the diversity of subspecialties in the field.

## Discussion

Our data revealed gaps in exposure to GI/Hep among medical students. While 75% (*N* = 15) of respondents were interested in pursuing a career with a procedural component, 60% (*N* = 14) had never had exposure to GI/Hep outside of the classroom. After this workshop, students endorsed increased familiarity with sub-specialties of GI/Hep, training pathways in GI/Hep, and daily lifestyles of different specialists in the field. Additionally, most respondents felt that the workshop changed their perception about the field of GI/Hep. Free response answers indicated that students learned about the diversity of subspecialties in the field, flexibility to carve a niche for oneself, and the positive culture of the field. Typically, these types of perspectives are more readily available to residents and fellows, and this workshop allowed an inside look into the nuances of careers in GI/Hep at an earlier training level.

This workshop had the highest level of interest and attendance from the MS1/MS2 classes. This suggests that early medical school learners have the desire and schedule flexibility to pursue experiences outside of the standard curriculum. Most respondents were in their MS2 year. The second year of medical school typically marks the transition from the basic sciences curriculum to clinical rotations [1]. Increased interest shown by the MS2 participants demonstrates a desire to explore various specialties and apply their basic sciences learning to clinical practice during this time. Simulation-based workshops serve as valuable tools to reinforce classroom-based learning as students transition to their clinical rotations [6]. Simulations have been shown to improve medical knowledge, comfort in procedures, and enhance teamwork and communication [5]. A study surveying members of a GI/Hep professional group revealed that the majority decided to pursue GI/Hep in medical school, and this decision was influenced primarily by mentors and hands-on training modules [7].

In addition, this panel and workshop provided an interactive, in-person experience for students. MS1 and MS2 students often lack engagement with trainees and faculty in medicine, given the separation between pre-clinical and clinical curricula. This format allows them to develop connections with residents, fellows, and faculty as they search for mentors. The importance of mentors in medicine is widely known to be a key career advancement tool [5]. Mentors can serve as role models that provide explicit academic knowledge about medicine as well as implicit knowledge about professionalism and values of the field [6]. The panel in our workshop introduced students to eminent faculty within GI/Hep who provided firsthand accounts of their experiences. Students could personally speak to these faculty members after the session or contact them afterwards for more information. A study examining the effects of an interactive medicine workshop revealed that the faculty panel was the main contributing factor in promoting interest among the students [8]. This is likely because firsthand experiences provide interesting narratives, and students are introduced to faculty who can serve as long-term guides for career development.

Traditionally, pre-clinical learning takes place in large group lecture settings. In addition, significant portions of pre-clinical curricula are offered virtually [9]. The Association of American Medical Colleges (AAMC) 2023 revealed that only 31.5% of respondents attended in-person lectures “most of the time” (AAMC) [10], suggesting that many students do not have regular in-person interactions with their peers or professors in an academic setting [9]. Given the reduced contact with peers and mentors during pre-clinical years, panel and simulation events such as this workshop can serve as engaging forums to connect students with like-minded peers. Other studies have aimed to introduce novel educational models to increase medical student engagement, such as the flipped classroom model [8], small-group active learning [11], gamification [12], and standardized patient encounters [13,14].

Our workshop utilized simulation exercises to increase medical student engagement; however, simulations can pose several challenges. One of these challenges includes sustaining student attention and motivation to participate. To address this concern, we simplified each simulation station to demonstrate a specific procedure or concept in GI/Hep within ten minutes before students switch to the next topic, keeping the momentum of learning through the workshop and interest from participants. In doing so, we were also able to tackle another challenge of simulation, which is the extensive preparation required. By keeping each station focused on specific topics, each station was able to have a standardized set of materials that could be moved to other locations, reused for multiple sessions, and can be available at different institutions.

Our study is not without limitations. Both this workshop and the surveys were optional for students, resulting in selection bias. This study also took place at a single institution with a relatively small cohort of students, limiting the generalizability of the data. We advertised through multiple email re-sends and messages to the medical student body and attracted an albeit small cohort of participants for the event. There are many reasons for this small sample size, including varied student schedules, conflicts with exam dates, and other concurrent extracurricular activities. However, the value this workshop provided for the students was evident in their survey responses. These results can help encourage and influence similar future endeavors across institutions and specialties. The students who attended the session uniformly enjoyed learning about careers in GI/Hep from the panelists, and they all stated that the simulations and procedural stations helped provide a greater understanding of the concepts discussed. 

So, the workshop we crafted did help us meet the objectives we had set out with, and this can provide a valuable template for other educational institutions. In future workshops, we aim to pay additional attention to improving outreach and recruitment of students. One idea is to raise awareness of the workshop during the medical school pathophysiology course when GI/Hep related topics are being covered, or during gastroenterology clerkships and electives. In the future, we shall also try to poll students who did not attend to find out which aspects of this workshop could be modified to appeal to a larger body of students. Additional studies with similar workshop formats are needed across specialties and institutions to assess the impact of this intervention on a larger scale. To assess long-term impact, attendees can also be followed over time to assess rates at which they sought out GI/Hep related electives, scholarly pursuits, and careers. 

## Conclusions

This workshop achieved the aims of introducing medical students to career paths in GI/Hep and demonstrating procedural applications of the concepts discussed. It incorporated aspects of mentorship by introducing students to internal medicine residents, GI/Hep fellows, and GI/Hep specialists who provided them with their own perspectives at various points in the training pathway through panel-based discussions. This workshop also incorporated simulation-based learning to teach students about procedures in GI/Hep. We believe continuing such sessions at our institution and others shall greatly augment learning and exposure for medical students and allow them to make early informed choices about career planning and strongly supplement traditional systems-based didactic curricula.

## Appendices

Appendix A 

**Table 2:** GI/Hep Workshop Pre-Survey

GI/Hep Workshop Pre-Survey
Please select your year in training: a. MS1 b. MS2 c. MS3 d. MS4
Have you had any prior exposure to the field of Gastroenterology (GI)/Hepatology (Hep) outside the classroom? a. Yes b. No
As per your understanding, which of these best describes GI/Hep? a. A medicine based subspecialty that includes procedures b. A surgical subspecialty c. A nonprocedural medicine based subspecialty
How interested are you in pursuing a career in GI/Hep from 1 to 5 (1 being not at all interested and 5 being extremely interested)?
How familiar are you with the following domains within GI/Hep (not at all, slightly, moderately, considerably, completely)? a. General endoscopy b. Advanced endoscopy c. Motility and neurogastroenterology d. Inflammatory Bowel Disease e. Liver medicine f. Transplant hepatology g. Hereditary cancer syndromes h. Medical education i. Immunology, Microbiome, and Metabiome Research
How familiar are you with the training pathway to be a GI/Hep from 1 to 5 (1 being not at all familiar and 5 being extremely familiar)?
How familiar are you with the day-to-day lifestyle of a GI/Hep specialist (1 being not at all familiar and 5 being extremely familiar)?
Are you interested in having a procedural component to your career? a. Yes b. No c. Maybe
Do you feel that medical school training should incorporate more networking events and career exposure during MS1/MS2 years? a. Yes b. No

Appendix B 

**Table 3:** GI/Hep Workshop Post-Survey

GI/Hep Workshop Post-Survey
Please select your year in training: a. MS1 b. MS2 c. MS3 d. MS4
As per your understanding, which of these best describes GI/Hep? a. A medicine based subspecialty that includes procedures b. A surgical subspecialty c. A nonprocedural medicine based subspecialty
How interested are you in pursuing a career in GI/Hep from 1 to 5 (1 being not at all interested and 5 being extremely interested)?
How familiar are you with the following domains within GI/Hep (not at all, slightly, moderately, considerably, completely)? a. General endoscopy b. Advanced endoscopy c. Motility and neurogastroenterology d. Inflammatory Bowel Disease e. Liver medicine f. Transplant hepatology g. Hereditary cancer syndromes h. Medical education i. Immunology, Microbiome, and Metabiome Research
How familiar are you with the training pathway to be a GI/Hep from 1 to 5 (1 being not at all familiar and 5 being extremely familiar)?
How familiar are you with the day-to-day lifestyle of a GI/Hep specialist (1 being not at all familiar and 5 being extremely familiar)?
Did today's session change your perception about the field of GI/Hep (if yes, please elaborate). a. Yes b. No
Please take a moment to rate your satisfaction with the following components of the workshop from 1 to 5 (with 1 being not at all satisfied and 5 being extremely satisfied). a. Advertising b. Panel Discussion c. Panel Composition d. Hands-on Simulations
Please provide any suggestions to improve this workshop. (optional)

## References

[1] Dornan T, Bundy C (2004). What can experience add to early medical education? Consensus survey. BMJ.

[2] Akhilesh Pathipati (The challenges of career planning in medical school. SCOPE Beyond the Headlines. Published online May 23). Akhilesh Pathipati. https://scopeblog.stanford.edu/2018/05/23/the-challenges-of-career-planning-in-medical-school/.

[3] Chang K, Panchal D, Bowman J, Sheikh S, Mohsin H (2023). Evaluating the impact of early career academic medicine workshops on medical students’ interest. Cureus.

[4] DeSipio J, Gaughan J, Perlis S, Phadtare S (2018). Use of real patients and patient-simulation-based methodologies for teaching gastroenterology to pre-clinical medical students. Healthcare (Basel).

[5] Okuda Y, Bryson EO, DeMaria S Jr, Jacobson L, Quinones J, Shen B, Levine AI (2009). The utility of simulation in medical education: what is the evidence?. Mt Sinai J Med.

[6] Gopal M, Skobodzinski AA, Sterbling HM, Rao SR, LaChapelle C, Suzuki K, Litle VR (2018). Bronchoscopy simulation training as a tool in medical school education. Ann Thorac Surg.

[7] Hann A, Lammert F, Walter S, Jansen PL (2019). Survey: Why did you become a gastroenterologist?. Z Gastroenterol.

[8] Frei E, Stamm M, Buddeberg-Fischer B (2010). Mentoring programs for medical students--a review of the PubMed literature 2000-2008. BMC Med Educ.

[9] Henry-Noel N, Bishop M, Gwede CK, Petkova E, Szumacher E (2019). Mentorship in medicine and other health professions. J Cancer Educ.

[10] Greenberg GS, Mansour M (2022). Evaluation of a novel cardiology undergraduate medical education curriculum. Cureus.

[11] (2023). Association of American Medical Colleges: Year Two Questionnaire (Y2Q) 2023. https://www.aamc.org/data-reports/students-residents/report/year-two-questionnaire-y2q.

[12] Krishnamurthy K, Selvaraj N, Gupta P (2022). Benefits of gamification in medical education. Clin Anat.

[13] Grijpma JW, Mak-van der Vossen M, Kusurkar RA, Meeter M, de la Croix A (2022). Medical student engagement in small-group active learning: a stimulated recall study. Med Educ.

[14] Williams DM, Bruggen JT, Manthey DE, Korczyk SS, Jackson JM (2020). The GI simulated clinic: a clinical reasoning exercise supporting medical students’ basic and clinical science integration. MedEdPORTAL.

